# Timing of nebulized colistimethate sodium following bronchodilator airway conditioning in hospital-acquired pneumonia: a target trial emulation study

**DOI:** 10.3389/fphar.2026.1859337

**Published:** 2026-09-18

**Authors:** Yi Cui, Qiannan Zhao, Jianning Zhang, Siyu Lu, Shuai Li, Lufeng Zhao, Ming Gao, Hao Chen, Hui Han, Xiaofang Huang

**Affiliations:** 1 Department of Critical Care Medicine, Qilu Hospital of Shandong University, Jinan, Shandong, China; 2 School of Nursing and Rehabilitation, Shandong University, Jinan, Shandong, China; 3 Innovation Research Center for Sepsis and Multiple Organ Injury, Shandong University, Jinan, Shandong, China; 4 Department of Respiratory and Intensive Care Unit, Shandong Institute of Respiratory Diseases, The First Affiliated Hospital of Shandong First Medical University and Shandong Provincial Qianfoshan Hospital, Jinan, Shandong, China; 5 Clinical Research Center of Shandong University, Clinical Epidemiology Unit, Qilu Hospital of Shandong University, Jinan, Shandong, China; 6 Department of Epidemiology and Health Statistics, School of Public Health, Cheeloo College of Medicine, Shandong University, Jinan, Shandong, China

**Keywords:** colistin, hospital-acquired pneumonia (HAP), microbiological clearance, multi-drug-resistant Gram-negative bacteria (MDR-GNB), target trial emulation

## Abstract

**Background:**

Evidence for nebulized colistimethate sodium (CMS) in hospital-acquired pneumonia (HAP) remains inconsistent, potentially due to heterogeneity in airway conditions and delivery timing. We evaluated whether timely initiation of nebulized CMS following bronchodilator airway pre-treatment was associated with microbiological outcomes in critically ill patients.

**Methods:**

We conducted a multicenter retrospective cohort study using target trial emulation among adult ICU patients with HAP who received nebulized CMS following bronchodilator nebulization. Two per-protocol strategies were compared: initiation within 90 min of bronchodilator nebulization (LE90) versus later initiation (GT90). Cloning, censoring, and inverse probability of censoring weighting were applied to address time-dependent confounding. The primary outcome was 14-day cumulative incidence of microbiological clearance, treating death as a competing event.

**Results:**

Among 218 eligible patients, timely initiation was associated with a higher weighted cumulative incidence of microbiological clearance at day 14 (71.9% vs. 52.6%; absolute difference 19.3%, 95% CI 4.8–32.5; *P* = 0.012) and a shorter restricted mean time alive and not microbiologically cleared (6.85 vs. 10.08 days; difference −3.23 days, 95% CI −4.30 to −2.08; *P* < 0.001). Findings were consistent across subgroups and robust in sensitivity analyses. No significant differences were observed in clinical success, superinfection, mortality, ICU length of stay, or ventilator-free days. Bronchospasm incidence was similar between strategies.

**Conclusion:**

Timely initiation of nebulized CMS within a defined post-bronchodilator window was associated with higher microbiological clearance and reduced persistent infection, without increased airway adverse events. These findings are hypothesis-generating and require prospective validation, particularly with respect to patient-centered outcomes.

## Graphical Abstract

**Figure:**
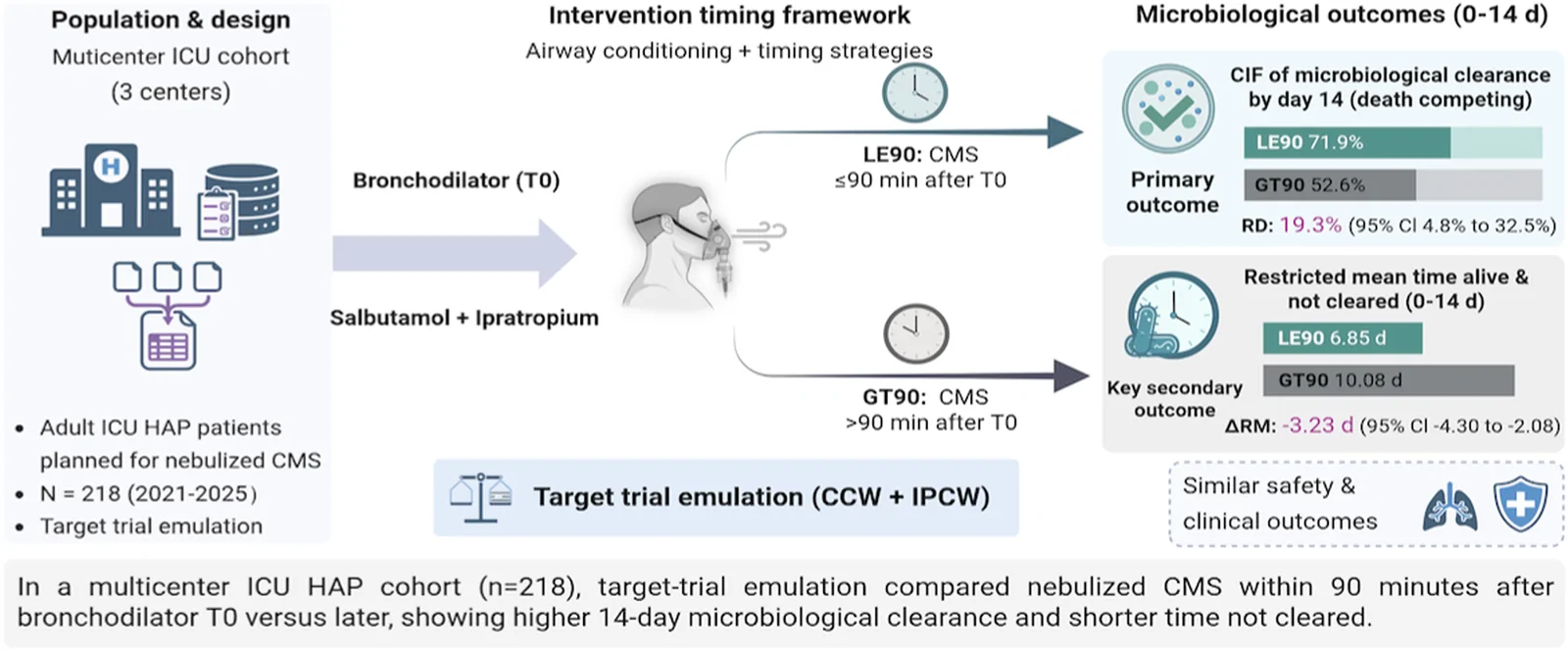
Created in BioRender. Cui, Y. (2026) https://BioRender.com/7lkxdde BioRender Academic License (Publication License; Agreement No. BH2A6JI8A6) Source: BioRender.com Status: Original graphical abstract created by the authors using BioRender.

## 1 Background

Hospital-acquired pneumonia (HAP) caused by multidrug-resistant Gram-negative bacteria (MDR-GNB) remain major challenges in the intensive care unit (ICU), being associated with high morbidity, prolonged mechanical ventilation, and excess mortality despite advances in systemic antimicrobial therapy (Zhang et al., 2026; Chang et al., 2021). Infections caused by carbapenem-resistant Enterobacteriaceae, *Acinetobacter baumannii* and *Pseudomonas aeruginosa* present significant clinical challenges. Polymyxin remains one of the last-line agents against MDR-GNB infections (Assefa, 2022); however, its efficacy is constrained by limited lung penetration and systemic toxicity associated with intravenous administration of antibiotics alone (Lee et al., 2024; Tuon et al., 2014).

Aerosolized colistimethate sodium (CMS, polymyxin E) has emerged as an important adjunctive therapy for MDR-GNB pneumonia, enabling high local antibiotic concentrations at the site of infection while minimizing systemic exposure and nephrotoxicity (Lee et al., 2024; Meschiari et al., 2024; Katip et al., 2024). Several clinical studies and meta-analyses have suggested that inhaled CMS may improve microbiological eradication or clinical response when added to intravenous therapy, especially in critically ill, mechanically ventilated patients (Liu et al., 2015; Haworth et al., 2024; Rattanaumpawan et al., 2010). However, the reported microbiological benefits remain heterogeneous, and optimal administration strategies for aerosolized CMS have not been clearly established (Vardakas et al., 2018). One potential explanation for the variable efficacy of inhaled antibiotics is impaired pulmonary drug delivery caused by bronchoconstriction and mucus plugging, which are commonly observed in critically ill patients with severe pneumonia (Labiris and Dolovich, 2003; Darquenne et al., 2024). Experimental and clinical data indicate that bronchodilator pre-treatment may improve aerosol deposition, enhance distal airway penetration, and reduce treatment-related bronchospasm (Usmani et al., 2005; Harrison and Laube, 1994). Consequently, international guidelines and expert consensus documents recommend the use of inhaled short-acting β_2_-agonists and/or anticholinergics prior to aerosolized antibiotic administration, is considered to reduce bronchospasm and optimize distal airway deposition (Palmer and Smaldone, 2014; Chalmers et al., 2025). In mechanically ventilated patients, in addition to airway conditions, aerosol delivery efficiency is also influenced by multiple factors, including nebulizer technology, circuit configuration and position, humidification, artificial airway characteristics, and ventilatory parameters (Zhang et al., 2026). Together, these considerations underscore the need for procedural and standardized delivery strategies to minimize avoidable losses during nebulized antibiotic administration. Nevertheless, few clinical studies have systematically evaluated whether a programmed, time-window–based sequential nebulization strategy, particularly following standardized airway pre-treatment, can translate into improved microbiological outcomes.

Against this background, we conducted a clinical study to investigate whether airway pre-treatment with aerosolized salbutamol plus ipratropium bromide, followed by a time-window–constrained sequential administration of aerosolized CMS, could improve microbiological outcomes in critically ill patients compared with delayed or non-programmed nebulization. We hypothesized that this strategy would increase the cumulative incidence of microbiological clearance within 14 days, accelerate pathogen eradication, and reduce the restricted mean time alive and not microbiologically cleared, while appropriately accounting for death as a competing event.

## 2 Methods

### 2.1 Study design and data source

We conducted a multi-center retrospective cohort study designed to emulate a per-protocol target trial of timing of nebulized CMS after bronchodilator nebulization in adult patients with HAP in the ICU, all of whom received a standardized nebulized CMS regimen of 3 MIU (∼90 mg colistin base activity) (Gkoufa et al., 2022; Andrade, 2025; Fu, 2023). Routinely collected electronic medical record data from three tertiary academic teaching hospitals with mixed medical-surgical ICUs were used. Consecutive eligible ICU admissions between 1 January 2021, and 30 June 2025, were screened.

Time zero (T_0_) was defined as the initiation of bronchodilator nebulization (salbutamol plus ipratropium bromide). The protocol, including eligibility criteria, strategy definitions, follow-up, outcomes, and the statistical analysis plan, was prespecified before model fitting within a target trial emulation framework. Reporting followed the Strengthening the Reporting of Observational Studies in Epidemiology (STROBE) guidelines, with explicit specification of key trial-emulation components (eligibility, strategies, assignment emulation, follow-up, estimand, and analytic methods (Cashin et al., 2025). The study was approved by the institutional review board of the coordinating center (Approval No. YXLL-KY-2026(048)), with additional site approvals as required. Informed consent was waived because this was a retrospective analysis of de-identified data.

### 2.2 Definition of HAP and MDR-GNB

HAP was defined as pneumonia occurring ≥48 h after hospital admission and not incubating at admission, diagnosed based on new or progressive pulmonary infiltrates on chest imaging plus at least two of the following: fever or hypothermia, leukocytosis or leukopenia, purulent respiratory secretions, or worsening oxygenation. MDR-GNB were defined as Gram-negative organisms that were non-susceptible to at least one agent in ≥3 antimicrobial classes, using *in vitro* susceptibility results from respiratory (or other clinically relevant) cultures obtained during the index HAP episode. For reporting, MDR-GNB were further categorized by pathogen group (e.g., carbapenem-resistant *A. baumannii*, carbapenem-resistant *P. aeruginosa*, and carbapenem-resistant *Enterobacterales*) according to local laboratory definitions of carbapenem non-susceptibility.

### 2.3 Eligibility criteria and strategy definition

Eligibility was assessed at T_0_. We included adult ICU patients with HAP who initiated bronchodilator nebulization and were eligible for nebulized CMS at T_0_, with sufficient treatment-timing and outcome timestamps for strategy assignment and follow-up. If multiple eligible ICU episodes were present, only the first episode was analyzed.

Patients were excluded if nebulized CMS was initiated before T0, or if key timestamps were implausible or nonreconstructable (e.g., negative treatment intervals). The primary per-protocol comparison contrasted two prespecified dynamic post-T0 initiation strategies: LE90 (CMS initiation within 90 min) and GT90 (no initiation within 90 min, i.e., initiation after 90 min). The 90-min window was prespecified to coincide with the expected peak bronchodilator response after nebulized salbutamol/ipratropium (Garrett et al., 1997; Karpel et al., 1990). As a prespecified sensitivity analysis, we repeated the full target trial emulation using a 120-min adherence window (LE120 vs. GT120), keeping all other analytic steps unchanged.

For descriptive baseline tables only, patients were grouped by observed initiation timing (≤90 min vs. >90 min); these observed groups were not interpreted as causal strategy groups.

### 2.4 Assignment emulation, cloning, and artificial censoring

Because treatment timing was not randomized, strategy assignment was emulated using clone-censor-weighting. At T_0_, each eligible patient was duplicated into two clones, one assigned to LE90 and one assigned to GT90 (Maringe et al., 2020).

For each clone, adherence was assessed during the 0- to 90-min grace period after T_0_. A clone was artificially censored at first protocol deviation: LE90 clones were censored at 90 min if CMS had not started by then; GT90 clones were censored at observed CMS start if CMS was initiated within 90 min. If an outcome event occurred before the planned censoring time, censoring was not applied. Follow-up then proceeded from T_0_ to the prespecified endpoint horizon.

This emulation targets the per-protocol contrast between timely and delayed initiation while reducing immortal-time and time-varying adherence biases inherent in naïve analyses of observed timing (Hernán and Robins, 2016).

### 2.5 Inverse probability of censoring weighting

Artificial censoring due to protocol deviation was addressed using inverse probability of censoring weighting (IPCW) (Maringe et al., 2020; Hernán et al., 2000). During the 0- to 90-min adherence window, follow-up was discretized into 5-min intervals, and interval-specific censoring probabilities were estimated with pooled logistic models separately within each strategy arm.

Stabilized inverse probability of censoring weights were constructed using an interval-only numerator model and a denominator model including interval plus prespecified baseline covariates measured at or before T_0_ (Maringe et al., 2020). Covariates were selected *a priori* based on clinical knowledge of factors associated with treatment timing and outcomes and included center, age, sex, body mass index (BMI), acute physiology and chronic health evaluation (APACHE) II score, sequential organ failure assessment (SOFA) score, chronic obstructive pulmonary disease (COPD), vasopressor use, mechanical ventilation status, PaO_2_/FiO_2_ ratio, HAP/VAP status, concomitant intravenous colistin, lactate level, and nebulization device type. To improve numerical stability under practical positivity constraints, stabilized IPCW were truncated at the 1st and 99th percentiles; as a prespecified stability safeguard, interval-level predicted censoring probabilities were bounded away from 0 to 1 (ε = 0.05) (Sr and Ma, 2008; Platt et al., 2012). Weight diagnostics (clipping proportion, truncation fraction, extreme weights, and effective sample size) are reported in Supplementary Table S1. As a prespecified sensitivity analysis, we repeated the full emulation using alternative IPCW specifications, increasing the probability bounds to ε = 0.10 (i.e., bounding to [0.10, 0.90]) and truncating stabilized weights at the 2.5th and 97.5th percentiles.

Missing baseline covariate values were infrequent and were handled using single imputation based on the sample mean (for continuous variables) prior to weight estimation. Because the proportion of missingness was low and imputation was performed before application of IPCW, this approach was not expected to materially influence effect estimates.

### 2.6 Outcomes and follow-up

The prespecified primary estimand was the between-strategy risk difference (RD; LE90 minus GT90) in the cumulative incidence of microbiological clearance by day 14, with death treated as a competing event (van Walraven and McAlister, 2016; Coemans et al., 2022). Microbiological clearance was determined exclusively from routine microbiology laboratory reports generated during clinical care, without investigator adjudication. Cumulative incidence functions (CIFs) were estimated under the inverse probability of censoring weighting framework described above.

The key secondary estimand was the between-strategy difference in restricted mean time alive and not microbiologically cleared through day 14 (ΔRM; LE90 minus GT90), estimated over the same 14-day horizon using weighted survival curves. Lower values indicate fewer days spent alive without microbiological clearance.

Secondary outcomes were clinical success by day 14, superinfection by day 28, all-cause mortality by day 28, ICU length of stay, ventilator-free days through day 28, and bronchospasm. Clinical success at day 14 was defined *a priori* as survival to day 14 with clinical improvement and without treatment escalation; death before day 14 or any escalation was classified as clinical failure. Superinfection by day 28 was defined as defined as a new episode of respiratory infection occurring after documented microbiological clearance of the index pathogen, with a positive respiratory culture yielding an organism distinct from the original causative pathogen and supported by compatible clinical evidence of infection. Secondary outcomes were analyzed using the same weighting framework when applicable.

Prespecified subgroup analyses for the primary and key secondary estimands were conducted by age (<65 vs. ≥65 years), BMI (<25 vs. ≥25 kg/m^2^; alternative 3-level coding in sensitivity analyses), SOFA (<8 vs. ≥8; alternative 3-level coding in sensitivity analyses), and COPD (yes vs. no). Subgroup effects were estimated within strata and assessed for heterogeneity using interaction terms.

In *post hoc* exploratory analyses restricted to patients who achieved microbiological clearance, landmark analyses were performed at 7 days after clearance to examine whether persistent ICU stay at the landmark was associated with subsequent mortality. Patients had to survive to the landmark and have ascertainable ICU status at that time. Additional exploratory analyses examined whether persistent ICU stay at day 7 after clearance was associated with superinfection by day 28.

### 2.7 Statistical analysis

All analyses followed the prespecified target trial emulation framework from T_0_. For the primary outcome, weighted cumulative incidence of microbiological clearance by day 14 was estimated using the Aalen-Johansen estimator with death as a competing event. The primary effect measure was the between-strategy risk difference (RD; LE90 minus GT90). For the key secondary outcome, restricted mean time alive and not microbiologically cleared through day 14 was estimated as the area under the survival function for remaining alive and not cleared, defined as S_0_(t) = 1−CIF_clear_(t)−CIF_death_(t), with effects reported as the between-strategy difference (ΔRM; LE90 minus GT90). Estimation incorporated inverse probability of censoring weights as described above.

Binary secondary outcomes were analyzed as weighted risk differences, and continuous outcomes as weighted mean differences. All effect estimates were obtained under the same weighting framework. Uncertainty was quantified using center-stratified nonparametric bootstrap resampling (B = 1,000). Each bootstrap replicate repeated the full analytic pipeline, including cloning, artificial censoring, IPCW estimation, and weighted outcome estimation. We report percentile-based 95% confidence intervals (CIs) and two-sided bootstrap P values.

For prespecified subgroup analyses, stratum-specific effects were estimated using the same causal pipeline, and heterogeneity was assessed with bootstrap interaction contrasts (difference in treatment effects between strata). No multiplicity adjustment was applied; secondary and subgroup analyses were interpreted as supportive or exploratory. Analyses were performed in R (R Foundation for Statistical Computing) with prespecified reproducible scripts.

## 3 Results

### 3.1 Study population, baseline characteristics, and weighting diagnostics

A total of 242 adult ICU patients with HAP who received nebulized CMS in conjunction with bronchodilator nebulization were screened. Of these, 24 were excluded because nebulized CMS was initiated before T_0_ (n = 19) or key treatment timestamps were missing/nonreconstructable (n = 5), leaving 218 patients in the analytic cohort (Figure 1).

**FIGURE 1:**
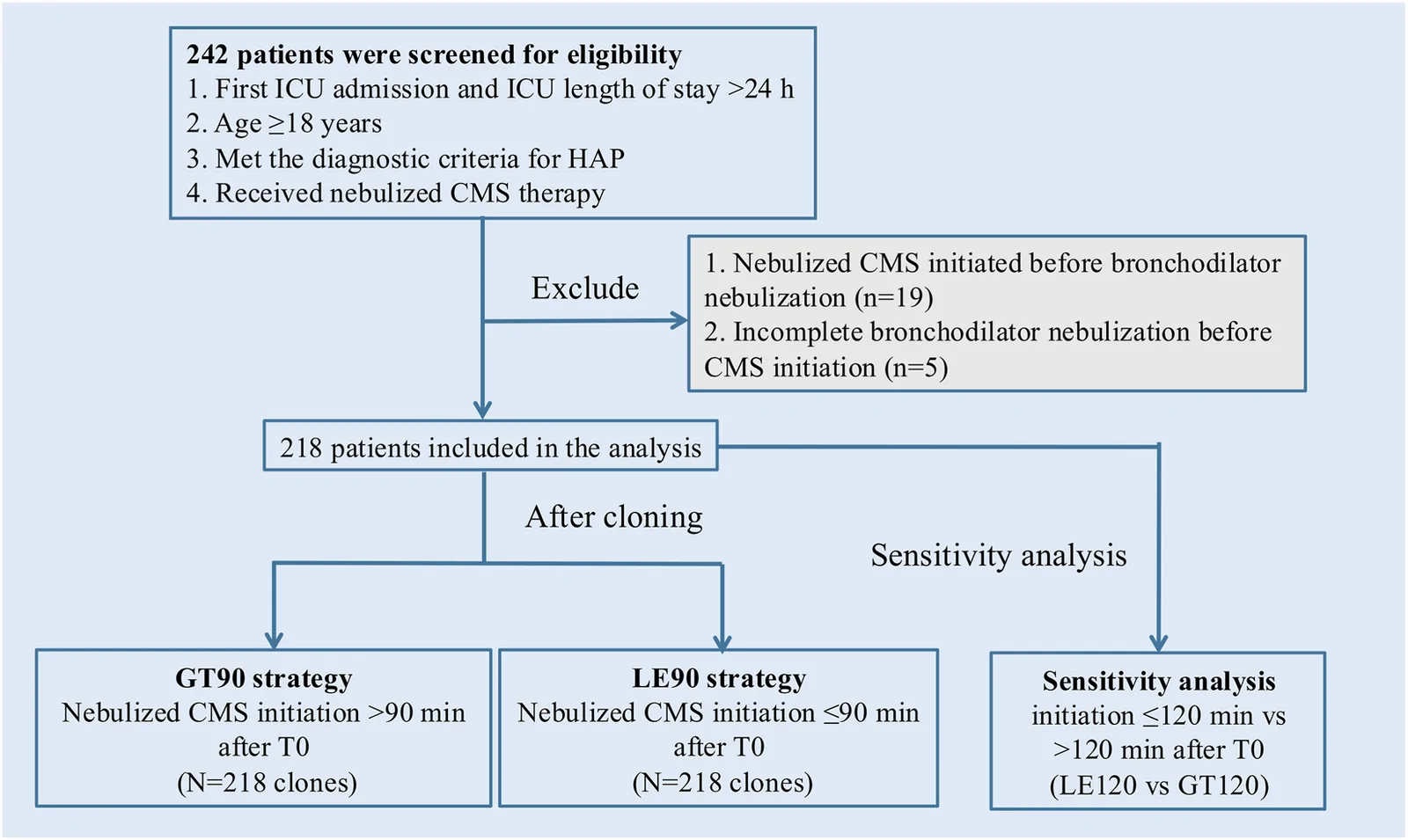
Patient selection and target trial emulation framework. A total of 242 adult ICU patients who met the diagnostic criteria for hospital-acquired pneumonia (HAP) and were intended to receive nebulized colistimethate sodium (CMS) were screened. 24 patients were excluded, including those who initiated nebulized CMS before bronchodilator nebulization (n = 19) and those with incomplete bronchodilator nebulization before CMS initiation (n = 5). The final analytic cohort included 218 patients. To emulate a target trial of post-bronchodilator initiation timing, each eligible patient was cloned at time zero (T_0_), defined as the start of bronchodilator nebulization, and assigned to both per-protocol strategies: initiation of nebulized CMS within 90 min after T0 (LE90) versus no initiation within 90 min after T_0_ (GT90). A prespecified sensitivity analysis used a 120-min window (LE120 vs. GT120). Abbreviations: CMS, colistimethate sodium; HAP, hospital-acquired pneumonia; ICU, intensive care unit; T_0_, time zero.

Baseline characteristics stratified by observed CMS initiation timing (≤90 min vs. >90 min) are summarized in Table 1. Among the 218 patients, 142 initiated CMS within 90 min and 76 initiated after 90 min. These observed timing groups are presented for descriptive purposes only and do not represent the analytically assigned strategies used in the target trial emulation. Missing data were present for selected physiologic variables (e.g., PaO_2_/FiO_2_, lactate, SOFA, and APACHE II), as detailed in Table 1. The distribution of causative pathogens, including carbapenem-resistant *A*. *baumannii*, *P*. *aeruginosa*, and *Enterobacterales*, was broadly similar across groups, although moderate imbalance was present overall (standardized mean difference [SMD] for pathogen category, 0.312).

**TABLE 1:** Baseline characteristics by observed timing of nebulized CMS initiation (≤90 min vs. >90 min)^a^.

Variable	Overall (N = 218)	Observed ≤90 min (N = 142)	Observed >90 min (N = 76)	Missing n (%)^b^	SMD^c^
Study center, n (%)					0.078
C01	100 (45.9%)	64 (45.1%)	36 (47.4%)	0 (0.0%)	—
C02	77 (35.3%)	52 (36.6%)	25 (32.9%)	0 (0.0%)	—
C03	41 (18.8%)	26 (18.3%)	15 (19.7%)	0 (0.0%)	—
Demographics					
Age, median [IQR]	66.00 [57.00, 76.00]	67.00 [59.00, 78.00]	63.00 [53.50, 70.50]	0 (0.0%)	0.383
Gender (male), n (%)	150 (68.8%)	96 (67.6%)	54 (71.1%)	0 (0.0%)	0.075
BMI, median [IQR]	25.20 [21.02, 29.10]	25.25 [21.22, 29.58]	25.05 [20.10, 28.10]	4 (1.8%)	0.162
VAP, n (%)	95 (43.6%)	65 (45.8%)	30 (39.5%)	0 (0.0%)	0.127
COPD, n (%)	76 (34.9%)	49 (34.5%)	27 (35.5%)	0 (0.0%)	0.021
Pathogens, n (%)					0.312
CRAB	105 (48.2%)	66 (46.5%)	39 (51.3%)	0 (0.0%)	—
CRPA	66 (30.3%)	46 (32.4%)	20 (26.3%)	0 (0.0%)	—
CRE	33 (15.1%)	25 (17.6%)	8 (10.5%)	0 (0.0%)	—
Others	14 (6.4%)	5 (3.5%)	9 (11.8%)	0 (0.0%)	—
Severity/physiology					
PaO_2_/FiO_2_, median [IQR]	190.00 [148.00, 238.00]	188.5 [148.00, 238.00]	194.00 [148.00, 236.75]	29 (13.3%)	0.089
Lactate, median [IQR]	2.16 [1.50, 2.70]	2.24 [1.63, 2.70]	2.00 [1.40, 2.52]	33 (15.1%)	0.218
SOFA, median [IQR]	7.50 [5.00, 10.00]	7.00 [5.00, 9.00]	8.00 [5.00, 10.00]	25 (11.5%)	0.078
APACHE II, median [IQR]	20.00 [16.00, 24.00]	19.00 [15.00, 23.00]	21.00 [17.00, 24.25]	22 (10.1%)	0.397
Device type (VMN), n (%)	144 (66.1%)	96 (67.6%)	48 (63.2%)	0 (0.0%)	0.093
Treatment					
Vasopressor use, n (%)	75 (34.4%)	47 (33.1%)	28 (36.8%)	0 (0.0%)	0.079
Mechanical ventilation, n (%)	162 (74.3%)	105 (73.9%)	57 (75.0%)	0 (0.0%)	0.024
Concomitant intravenous colistin, n (%)	23 (10.6%)	16 (11.3%)	7 (9.2%)	0 (0.0%)	0.068

^a^ Groups are based on observed initiation timing and are distinct from the per-protocol strategy assignment used in target trial emulation.

^b^ Missing n (%) is calculated from the original non-imputed baseline dataset.

^c^ For categorical variables with >2 levels, SMD, was defined as the maximum absolute SMD, across indicator levels.

Most baseline variables were generally comparable; however, nontrivial imbalance was observed for age and APACHE II score (SMDs approximately 0.38–0.40), suggesting potential baseline confounding in naive comparisons. In contrast, center distribution, sex, COPD, vasopressor use, mechanical ventilation status, nebulization device type, and concomitant intravenous colistin use showed relatively small between-group differences.

After weighting, covariate balance improved substantially (Supplementary Figure S1). Absolute standardized mean differences for all prespecified covariates were reduced to <0.10, supporting improved exchangeability between strategies. Weight diagnostics suggested acceptable performance of the IPCW models (Supplementary Figure S2): stabilized weights were centered near 1.0 with limited extremes after truncation; truncated weights were infrequent (LE90: 2.8%; GT90: 2.6%), and effective sample sizes remained adequate (LE90 ESS = 133.0; GT90 ESS = 71.3).

### 3.2 Primary and key secondary outcomes

Under the per-protocol emulated strategies, timely initiation of nebulized CMS within 90 min after bronchodilator nebulization (LE90) resulted in improved microbiological outcomes compared with no-initiation within 90 min (GT90) (Table 2).

**TABLE 2:** Primary and key secondary outcomes under per-protocol emulated strategies of timely versus delayed initiation of nebulized colistimethate sodium after bronchodilator nebulization.

Outcome (0-14 days)^a^	LE90	GT90	Effect (LE90-GT90)^b^	*P* value
Cumulative incidence of microbiological clearance by day 14, % (95% CI) (death treated as a competing event)	71.9 (63.6, 78.9)	52.6 (40.8, 64.9)	19.3 (4.8, 32.5)	0.012
Restricted mean time alive and not microbiologically cleared, days (95% CI)	6.85 (6.10, 7.62)	10.08 (9.24, 10.85)	−3.23 (−4.30, −2.08)	<0.001

^a^ Time zero (T_0_) was the start of bronchodilator nebulization. Per-protocol emulated strategies were: LE90, initiation of nebulized CMS within 90 min after T_0_; and GT90, no initiation within 90 min (i.e., initiation after 90 min or no initiation within 90 min). Effects were estimated using target trial emulation with clone–censor–weighting and IPCW from 5-min pooled logistic models with prespecified baseline covariates over the 0–90-min adherence window. Stabilized weights were truncated at the 1st/99th percentiles in the primary analysis. The day-14 cumulative incidence was estimated using the Aalen–Johansen estimator with death as a competing event. The restricted mean time alive and not microbiologically cleared (0–14 days) was defined as the area under $S_{0}\left(t\right)$ , where $S_{0}\left(t\right)=1-{\text{CIF}}_{\text{clear}}\left(t\right)-{\text{CIF}}_{\text{death}}\left(t\right)$ . 95% CIs and P values were obtained via center-stratified bootstrap resampling (B = 1,000), repeating the full analytic pipeline in each replicate.

^b^ Effects are reported as LE90−GT90. For restricted mean time alive and not cleared, negative values indicate lower burden (fewer days alive and not cleared) under LE90.

Abbreviations: CI, confidence interval; CMS, colistimethate sodium; GT90, no CMS, initiation within 90 min after T_0_; IPCW, inverse probability of censoring weighting; LE90, CMS, initiation within 90 min after T_0_; T_0_, start of bronchodilator nebulization.

By day 14, the weighted cumulative incidence of microbiological clearance (with death as a competing event) was 71.9% (95% CI, 63.6%–78.9%) in LE90% and 52.6% (95% CI, 40.8%–64.9%) in GT90, yielding an absolute risk difference of 19.3% (95% CI, 4.8%–32.5%; *P* = 0.012). For the key secondary estimand, the restricted mean time alive and not microbiologically cleared through day 14 was 6.85 days (95% CI, 6.10–7.62) in LE90 versus 10.08 days (95% CI, 9.24–10.85) in GT90, corresponding to −3.23 days (95% CI, −4.30 to −2.08; *P* < 0.001), indicating a lower cumulative burden of persistent infection with timely initiation.

Sensitivity analyses supported the robustness of these findings across alternative protocol and weighting specifications. Using a 120-min adherence window (LE120 vs. GT120), results remained directionally consistent (Supplementary Table S3). The weighted cumulative incidence of microbiological clearance by day 14 was 69.0% (95% CI, 61.0%–76.6%) under LE120% and 53.7% (95% CI, 40.7%–65.6%) under GT120, corresponding to an absolute risk difference of 15.5% (95% CI, 0.7%–30.6%; *P* = 0.046). The restricted mean time alive and not microbiologically cleared through day 14 was 7.16 days (95% CI, 6.42–7.95) under LE120 and 9.98 days (95% CI, 9.14–10.89) under GT120, yielding a difference of −2.84 days (95% CI, −4.01 to −1.64; *P* < 0.001). Similarly, under a more conservative IPCW specification (ε = 0.10; truncation at the 2.5th/97.5th percentiles), effect estimates were nearly identical to the primary analysis (Supplementary Table S4). The weighted cumulative incidence of microbiological clearance was 71.5% (95% CI, 63.7%–78.3%) under LE90% and 52.6% (95% CI, 41.5%–64.0%) under GT90 (absolute risk difference 18.6%, 95% CI, 5.8%–32.8%; *P* = 0.004), and the restricted mean time difference was −3.20 days (95% CI, −4.27 to −2.13; *P* < 0.001).

Respiratory culture surveillance intensity during follow-up was comparable between strategies (Supplementary Table S2). The median number of respiratory cultures obtained within 14 days was identical between groups (5 [IQR, 4–5] in LE90 vs. 5 (Tuon et al., 2014) in GT90). All patients had at least one follow-up respiratory culture after T0, and the timing of the first follow-up culture was similarly distributed across groups (SMD = 0.191). The proportion undergoing bronchoscopy during follow-up was also comparable (SMD = 0.112). Overall, standardized mean differences for surveillance-related variables were below commonly accepted imbalance thresholds, reducing concern that differential sampling intensity alone accounted for the observed differences in microbiological clearance.

### 3.3 Temporal patterns of microbiological clearance

Figure 2 depicts the weighted CIFs for microbiological clearance over the 14-day follow-up period under the per-protocol emulated strategies, with death treated as a competing event. Separation between the curves emerged early after bronchodilator nebulization and widened progressively over time, with consistently higher cumulative incidence observed under the timely initiation strategy (LE90) compared with no initiation within 90 min (GT90).

**FIGURE 2:**
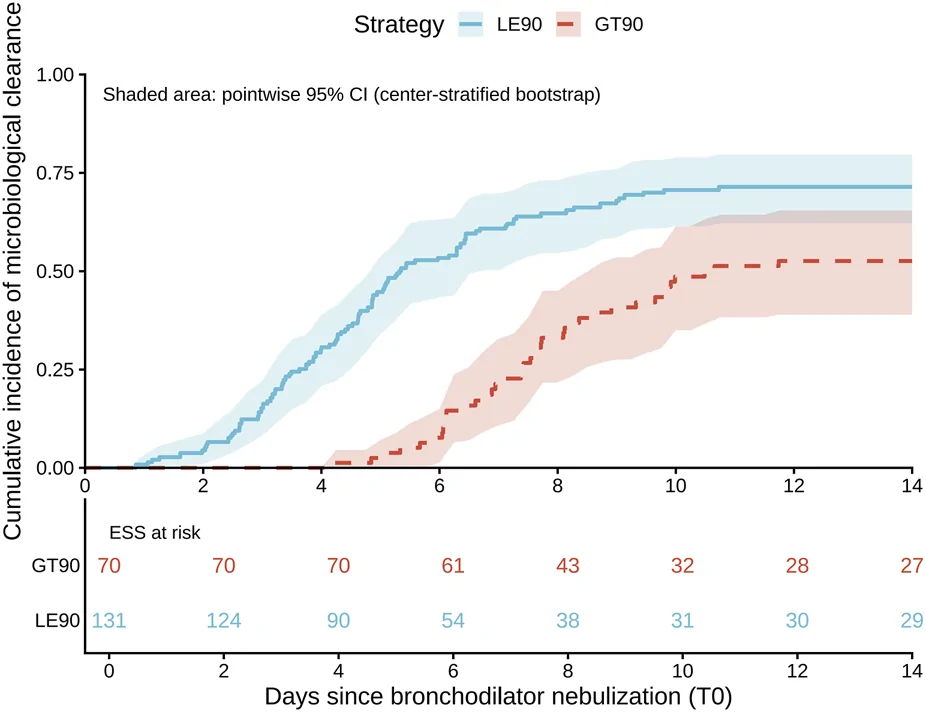
Cumulative incidence of microbiological clearance by day 14 under per-protocol emulated strategies. Shown are the cumulative incidence functions (CIFs) of microbiological clearance from day 0 to day 14 under the per-protocol emulated strategies of timely initiation (LE90) and delayed initiation (GT90) of nebulized colistimethate sodium following bronchodilator nebulization. Time zero (T_0_) was defined as the initiation of bronchodilator nebulization. Death was treated as a competing event. Shaded areas represent pointwise 95% confidence intervals derived from center-stratified bootstrap resampling. Numbers at risk indicate the effective sample size (ESS) under inverse probability of censoring weighting at each time point.

The difference became apparent within the first week and persisted through day 14, consistent with earlier and sustained microbiological clearance with timely initiation. Pointwise 95% CIs showed limited overlap during the mid-to late-follow-up period, although inference was based on the prespecified effect estimates rather than visual inspection alone. Effective sample sizes under inverse probability of censoring weighting remained stable throughout follow-up, indicating that weighted estimates were not driven by progressive weight instability over time.

### 3.4 Burden of persistent infection among survivors

Figure 3 illustrates the weighted probability of remaining alive and not microbiologically cleared over the 14-day follow-up period. Patients managed under the timely initiation strategy (LE90) experienced a more rapid decline in this probability compared with those under the no initiation within 90 min strategy (GT90), consistent with earlier and more frequent clearance events.

**FIGURE 3:**
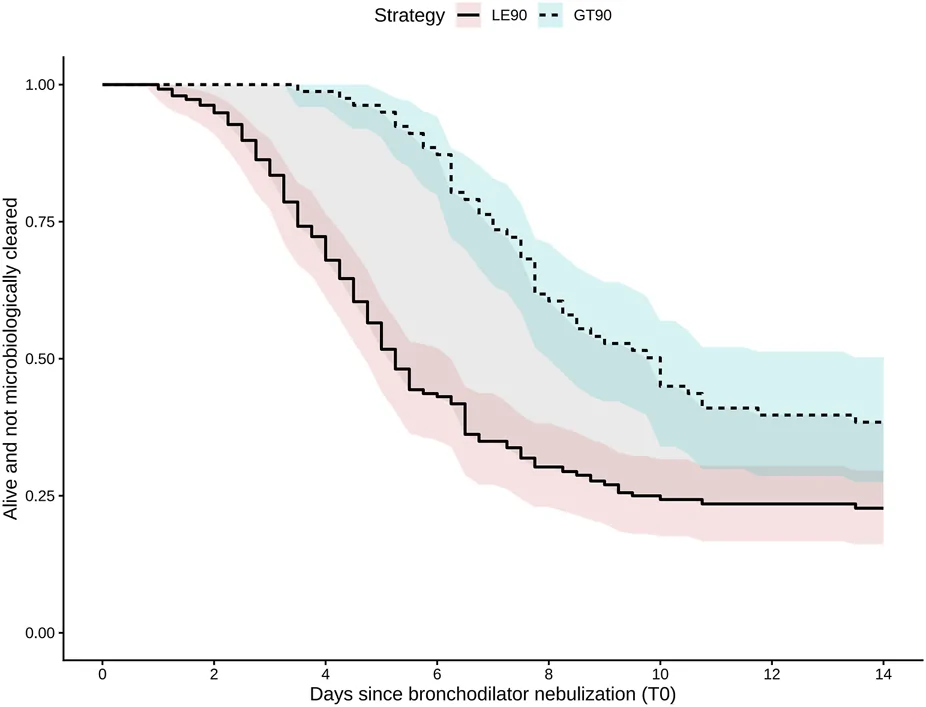
Probability of being alive and not microbiologically cleared by day 14 under per-protocol emulated strategies. State-occupation probability curves for being alive and not microbiologically cleared from day 0 to day 14 are shown for the timely-initiation strategy (LE90) and delayed-initiation strategy (GT90) of nebulized colistimethate sodium after bronchodilator nebulization. Time zero (T_0_) was defined as bronchodilator nebulization start. Shaded bands indicate pointwise 95% confidence intervals from center-stratified bootstrap resampling. The area under each curve corresponds to the restricted mean time alive and not microbiologically cleared over 14 days. The shaded region denotes the between-strategy difference in state-occupation probability at each time point; the integral of this shaded area over 0–14 days equals the difference in restricted mean time alive and not microbiologically cleared (ΔRM, in days).

The separation between curves emerged within the first few days after bronchodilator nebulization and persisted throughout follow-up, resulting in a correspondingly smaller area under the curve for the LE90 strategy. This graphical pattern corresponds to the reduced restricted mean time alive and not microbiologically cleared observed in the key secondary analysis, indicating a lower cumulative burden of persistent infection over time under timely initiation.

### 3.5 Secondary clinical and safety outcomes

Secondary clinical outcomes under the per-protocol emulated strategies are summarized in Table 3. In weighted analyses, clinical success by day 14 and all-cause mortality by day 28 showed effect estimates close to null, with confidence intervals crossing zero, indicating no statistically significant difference between the LE90 and GT90 strategies. Superinfection by day 28 occurred 5.9% more frequently in the LE90 group than in the GT90 group (95% CI −18.6%, 6.9%; *P* = 0.408). ICU length of stay and ventilator-free days through day 28 were comparable between the two groups, without evidence of a clinically meaningful difference.

**TABLE 3:** Secondary outcomes under per-protocol LE90 vs. GT90 initiation strategies.

Secondary outcome^a^	LE90	GT90	Effect (LE90 − GT90) Risk difference or mean difference (95% CI)^b^	*P* value
Clinical success by day 14	69.8%	65.3%	4.5% (−7.9%, 18.5%)	0.462
Superinfection by day 28	21.1%	27.0%	−5.9% (−18.6%, 6.9%)	0.408
All-cause mortality by day 28	24.7%	26.4%	−1.8% (−13.4%, 9.6%)	0.728
ICU length of stay, days	12.14	11.74	0.40 (−1.44, 2.40)	0.604
Ventilator-free days through day 28, days	13.33	11.64	1.69 (−0.90, 4.20)	0.198
Bronchospasm (adverse event)	3.8%	5.7%	−1.9% (−8.6%, 3.8%)	0.574

^a^ Time zero (T_0_) was defined as the start of the first bronchodilator nebulization. Two per-protocol strategies were compared: LE90 (initiation of nebulized CMS, within 90 min after T_0_) and GT90 (no initiation within 90 min). Effects were estimated using a target trial emulation with the clone–censor–weighting framework. IPCW, were estimated separately within each strategy using 5-min pooled logistic models adjusting for prespecified baseline covariates over the 0–90-min adherence window. Predicted censoring probabilities were clipped to 0.05, 0.95, and stabilized weights were truncated at the 1st and 99th percentiles. Clinical success at day 14 was defined as being alive at day 14, with clinical improvement, and without therapy escalation. Therapy escalation was defined as any addition or broadening of antibiotics, or intensification of organ support (e.g., increased ventilatory or vasopressor support) due to clinical deterioration. Death before day 14 or any therapy escalation was classified as clinical failure.

^b^ Effect estimates denote differences (LE90 − GT90). For binary outcomes, effects are risk differences; for continuous outcomes, effects are mean differences.

Abbreviations: CI, confidence interval; CMS, colistimethate sodium; CCW, clone–censor–weighting; GT90, initiation of nebulized CMS >90 min after T_0_ or no initiation within 90 min; ICU, intensive care unit; IPCW, inverse probability of censoring weighting; LE90, initiation of nebulized CMS ≤90 min after T_0_; T_0_, time zero.

Importantly, the incidence of bronchospasm did not differ between strategies, indicating that timely initiation of nebulized CMS following bronchodilator pre-treatment was not associated with an increased risk of airway-related adverse events.

### 3.6 Exploratory post-clearance analyses

To further characterize post-clearance clinical trajectories, exploratory landmark analyses were performed among patients who achieved microbiological clearance. At the 7-day landmark after clearance, 47 patients remained in the ICU and 84 had already left the ICU. Subsequent 28-day mortality was higher among those who remained in the ICU than among those who had left the ICU (31.9% vs. 10.7%); in multivariable Cox analysis, persistent ICU stay was associated with a significantly higher subsequent mortality risk (HR 3.35, 95% CI 1.37–8.18; *P* = 0.008) (Supplementary Table S5).

By contrast, LE90 was not clearly associated with earlier ICU liberation after clearance. At day 7 after clearance, 41 of 106 patients (38.7%) in the LE90 group and 12 of 41 patients (29.3%) in the GT90 group remained in the ICU, corresponding to a risk difference of 9.4% (95% CI, −7.3%–26.1%; Fisher’s exact *P* = 0.341) (Supplementary Table S6). In an additional exploratory analysis, patients who remained in the ICU at day 7 after clearance had a higher proportion of superinfection by day 28 than those who had left the ICU (42.6% vs. 20.2%; risk difference 22.3%, 95% CI, 5.8%–38.9%; *P* = 0.001) (Supplementary Table S7).

### 3.7 Subgroup analyses

Subgroup analyses for both prespecified estimands are shown in Figure 4. For the day-14 microbiological clearance risk difference, effects estimands were directionally consistent across strata, and no statistically significant interaction was detected. For the key secondary estimand (difference in restricted mean time alive and not microbiologically cleared), a statistically significant interaction was observed only for the SOFA subgroup, whereas interaction tests for other prespecified subgroups were not statistically significant. The magnitude of effect varied across SOFA strata; however, estimates remained directionally aligned with the overall treatment effect. Overall, subgroup findings were generally consistent with the main treatment effect pattern. The observed SOFA-related heterogeneity for the ΔRM endpoint should be interpreted cautiously given the exploratory nature of subgroup analyses and absence of multiplicity adjustment.

**FIGURE 4:**
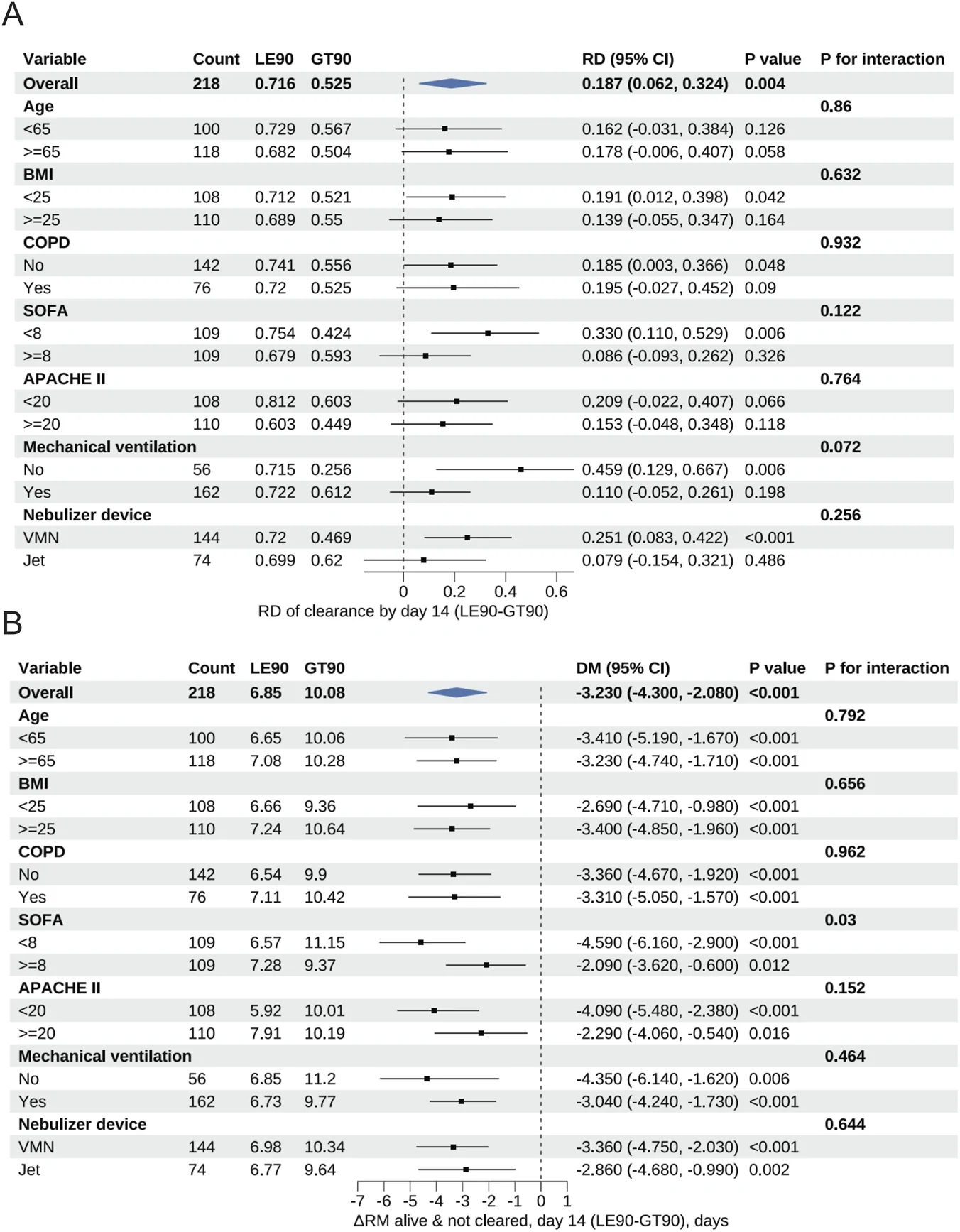
Subgroup analyses of the primary and key secondary outcomes under per-protocol emulated strategies. **(A)** Presents subgroup-specific risk differences (RDs) for microbiological clearance by day 14, comparing timely initiation (LE90) versus delayed initiation (GT90) of nebulized colistimethate sodium after bronchodilator nebulization. **(B)** Presents subgroup-specific differences in restricted mean time alive and not microbiologically cleared through day 14 (ΔRM, days); negative values favor LE90 (lower burden). Effect estimates and 95% confidence intervals were obtained using the same clone–censor–weighting and inverse probability of censoring weighting approach as in the primary analysis. Interaction *P* values assess heterogeneity of treatment effects across subgroups.

## 4 Discussion

In this target trial emulation of critically ill adults with HAP who received nebulized CMS in conjunction with bronchodilator nebulization, we demonstrate that initiation of nebulized CMS within a defined post-bronchodilator time window is associated with a substantially higher cumulative incidence of microbiological clearance within 14 days, accounting for death as a competing event, and a marked reduction in the cumulative burden of persistent infection among survivors, as quantified by the restricted mean time alive and not microbiologically cleared. These microbiological benefits were robust across clinically relevant subgroups, were not accompanied by an increased risk of bronchospasm, and were not associated with detectable differences in short-term mortality or global clinical outcomes.

The 90-min initiation window was prespecified before data analysis rather than selected empirically. It was chosen based on the expected pharmacodynamic profile of nebulized salbutamol plus ipratropium bromide, which generally achieves maximal or near-maximal bronchodilation within approximately 60–90 min while maintaining clinically meaningful airway smooth muscle relaxation. However, no direct pharmacodynamic studies have established that this interval corresponds to optimal pulmonary deposition or antibacterial efficacy of nebulized CMS. Therefore, the 90-min window should be regarded as a biologically informed operational definition rather than a validated physiological threshold. Reassuringly, prespecified sensitivity analyses using a 120-min initiation window yielded directionally consistent results, supporting the robustness of the observed association across clinically plausible time windows.

We selected microbiological clearance as the primary endpoint because the intervention under investigation is expected to exert its most direct effect by enhancing pulmonary antibiotic delivery and promoting bacterial eradication, rather than by immediately improving mortality or other complex patient-centered outcomes. In critically ill patients, mortality, ICU length of stay, and other clinical outcomes are influenced by numerous competing factors, including baseline disease severity, organ dysfunction, host immune responses, and noninfectious complications. Consequently, microbiological clearance represents the most proximal outcome expected to respond to optimization of nebulized antibiotic delivery, whereas improvements in downstream clinical outcomes are likely to require larger sample sizes or randomized trials to demonstrate.

The role of nebulized antibiotics in HAP and ventilator-associated pneumonia remains controversial, with international guidelines recommending selective rather than routine use due to heterogeneous efficacy data and concerns regarding safety and delivery efficiency (Tamma et al., 2024; Andre et al., 2016; Rello et al., 2017). Prior randomized trials and meta-analyses of nebulized antibiotics, including CMS, have reported more consistent improvements in microbiological eradication than in mortality or other hard clinical endpoints, with substantial between-study variability (Katip et al., 2024; Rattanaumpawan et al., 2010; Tang et al., 2021; Honore et al., 2024; Valachis et al., 2015). Our findings are concordant with this literature and suggest that variability in initiation timing may partly explain the inconsistent microbiological effects reported across studies. Accordingly, our results should not be interpreted as supporting routine use of nebulized CMS in all patients with HAP. Rather, they suggest that when nebulized CMS is already used in clinical practice, timely initiation within a defined post-bronchodilator window may represent potentially important determinants of microbiological effectiveness.

Importantly, microbiological clearance in critically ill patients is intrinsically a time-dependent event that competes with death, which may preclude the observation of bacterial eradication. Conventional survival or binary analyses may therefore introduce bias when assessing antimicrobial efficacy in this setting (Schuster et al., 2020; Lacny et al., 2018). Competing risk methods using CIFs, as well as restricted mean survival time–based metrics, provide a more clinically interpretable and robust framework to evaluate microbiological outcomes while accounting for early mortality (Mozumder et al., 2021; Resche-Rigon et al., 2006; Wolbers et al., 2014; Royston and Parmar, 2013; Kim et al., 2017). The early and sustained separation of weighted cumulative incidence curves observed in our study supports the biological plausibility of a timing-sensitive treatment effect.

Because microbiological outcomes depend on surveillance practices, differential sampling intensity could potentially bias clearance estimates. However, respiratory culture frequency during follow-up was comparable between strategies, with similar culture frequency, specimen types, bronchoscopy utilization and overall timing of follow-up cultures. Notably, the delayed-initiation group underwent the first follow-up culture earlier than the timely-initiation group, which argues against systematic overestimation of clearance in the LE90 strategy due to more intensive monitoring. Together, these observations make differential ascertainment an unlikely explanation for the observed difference in microbiological clearance. Nevertheless, because microbiological surveillance was not protocolized across participating centers, some degree of ascertainment bias cannot be completely excluded.

A key challenge in aerosolized antimicrobial therapy in ICU patients with HAP, especially ventilator-associated pneumonia, is the highly variable and often low lung deposition achieved during mechanical ventilation, influenced by airway resistance, secretion burden, ventilator settings, humidification, and device characteristics. Bronchoconstriction and uneven ventilation can further impair distal airway delivery (Montigaud et al., 2019; Dhanani et al., 2016; Dugernier et al., 2017). Although bronchodilator pre-treatment is commonly recommended to reduce bronchospasm and improve tolerability of inhaled antibiotics, outcome-level evidence supporting this practice has been limited (Chalmers et al., 2025; Terpstra et al., 2023). Our results suggest that bronchodilator pre-treatment, when coupled with timely execution of nebulized CMS, may create a clinically actionable pulmonary therapeutic window during which effective lung exposure is more likely to be achieved. The early and sustained separation of cumulative incidence curves supports this interpretation and is biologically plausible in light of prior work demonstrating that aerosol delivery efficiency is highly sensitive to administration conditions and technique (Dhanani et al., 2016; Dugernier et al., 2017). Importantly, the similar incidence of bronchospasm between strategies indicates that bronchodilator pre-treatment followed by timely nebulization was safe.

The lack of detectable differences in mortality, ICU length of stay, or ventilator-free days should be interpreted in the context of the multifactorial determinants of outcome in ICU pneumonia and is concordant with similar dissociations between microbiological and mortality outcomes observed in prior studies of nebulized antibiotics (Tang et al., 2021; Dugernier et al., 2017). In severe HAP caused by MDR-GNB, mortality is often driven by baseline illness severity, organ dysfunction, and noninfectious complications (Melsen et al., 2013; Papazian et al., 2020); the pathological cascade is typically well established before effective microbiological clearance can be achieved, such that timely bacterial eradication may function more as arresting ongoing bacterial injury than reversing already-established organ damage. In the present cohort, with a median baseline SOFA score of 7.5, a substantial proportion of patients likely had mortality trajectories driven predominantly by noninfectious factors at the time microbiological clearance was achieved. The statistically significant interaction observed in SOFA subgroup analyses for the key secondary estimand provides indirect support for this interpretation, suggesting that patients with greater physiological reserve may be more likely to translate microbiological benefit into detectable clinical improvement. From a statistical standpoint, the 28-day mortality difference of and the directionally consistent clinical success rate both reflect the limited power of the present cohort to detect differences of this magnitude rather than evidence of a null effect. From a clinical perspective, however, reducing the duration of persistent infection may still be meaningful by facilitating de-escalation, limiting antibiotic exposure, and potentially reducing downstream complications, even if a mortality signal is not demonstrable.

Our exploratory post-clearance landmark analyses further suggest that microbiological clearance should not be equated with clinical recovery. Among patients who achieved clearance, those who remained in the ICU at 7 days after clearance had a substantially higher subsequent mortality risk than those who had already left the ICU. Persistent ICU stay at day 7 was also associated with a higher crude proportion of superinfection, although adjusted estimates were unstable because of limited sample size and missing data. Together, these findings suggest that pathogen eradication alone may be insufficient to reverse the broader physiologic vulnerability of critically ill patients. One possible explanation is that elimination of the dominant MDR pathogen does not fully restore host defense or airway homeostasis and may, in some cases, create an ecological niche subsequently occupied by other organisms, including colistin-insusceptible bacteria, fungi, or Gram-positive pathogens. Such post-clearance vulnerability may contribute to late adverse outcomes and could partly attenuate the net survival benefit of targeted microbiological clearance.

A key strength of this study is its use of target trial emulation to compare time-specific initiation strategies, effectively addressing immortal time bias and time-dependent confounding found in simple early-versus-late comparisons. By explicitly emulating treatment assignment at time zero using cloning, artificial censoring, and inverse probability of censoring weighting, our analytic framework substantially reduces immortal-time bias and informative censoring that would arise in conventional early-versus-late comparisons. This approach enhances causal interpretability in real-world data when randomized trials are not possible (Hernán et al., 2022). The use of cloning, censoring, and inverse probability weighting is particularly suited to evaluating initiation windows that are indistinguishable at baseline (Webster-Clark et al., 2025; Hernán, 2018). Nevertheless, although this design substantially reduces bias from measured time-dependent confounding, it cannot completely eliminate residual confounding inherent to observational research, including potential bias due to model misspecification or unmeasured determinants of treatment adherence. Sensitivity analyses using a 120-min window yielded estimates in the same direction as the primary analysis, but with a smaller effect size, supporting qualitative robustness while suggesting a time-dependent attenuation.

Several limitations merit consideration. First, HAP requiring nebulized CMS represents a relatively selected and clinically specific population, inherently limiting achievable sample size in single-country, real-world cohorts. Although our study leveraged multicenter data and causal emulation methods, the modest cohort size may have limited statistical power for detecting smaller differences in hard clinical endpoints, underscoring the need for larger, multicenter studies or prospective randomized trials to confirm these findings. Second, residual confounding cannot be fully excluded despite emulation and weighting, particularly from unmeasured factors such as secretion burden, ventilator mode, humidification settings, airway resistance, workflow constraints and respiratory therapist–dependent nebulization practices, which may influence both timing adherence and outcomes. These factors may affect pulmonary deposition of nebulized antibiotics and therefore cannot be excluded as alternative explanations for part of the observed association. Accordingly, our findings should be interpreted as demonstrating an association rather than proving a direct causal physiological effect. Finally, bronchodilator dosing was not standardized across centers, reflecting routine clinical practice; however, the study focused on the timing of CMS initiation following airway preparation rather than the bronchodilator regimen itself. These findings suggest that the effectiveness of nebulized antibiotics in ICU pneumonia may depend more on their delivery method and timing than on their mere prescription. Our findings should be interpreted in the context of the retrospective observational design, the modest sample size, and the potential for residual and unmeasured confounding. Although timely CMS initiation was associated with improved microbiological outcomes, no statistically significant differences were observed in mortality, clinical success, ICU length of stay, or ventilator-free days. Therefore, the present findings do not establish that a defined initiation window improves patient-centered outcomes. A standardized workflow incorporating airway pre-treatment and timely CMS administration may be considered a hypothesis for prospective evaluation, but its clinical effectiveness and safety should be confirmed in larger multicenter cohorts or randomized studies.

## 5 Conclusion

In critically ill patients with HAP, timely initiation of nebulized CMS within 90 min after bronchodilator airway pre-treatment was associated with a higher cumulative incidence of microbiological clearance, accounting for death as a competing event, and a reduced cumulative burden of persistent infection over 14 days, without evidence of increased airway adverse events. These findings are hypothesis-generating, and prospective studies are needed to determine whether delivery timing influences patient-centered outcomes.

## Data Availability

The original contributions presented in the study are included in the article/Supplementary Material, further inquiries can be directed to the corresponding authors.
