# Supplementary material for: Timing of nebulized colistimethate sodium following bronchodilator airway conditioning in hospital-acquired pneumonia: a target trial emulation study
Source: Front Pharmacol. 2026 Sep 18;17:1859337. doi: 10.3389/fphar.2026.1859337 (PMC13630638; doi:10.3389/fphar.2026.1859337)
Supplement: Supplementary file 1 [file Supplementaryfile1.docx]

**Timing of nebulized colistimethate sodium following bronchodilator airway conditioning in hospital-acquired pneumonia: a target trial emulation study**

Yi Cui^1,2,3†^, Qiannan Zhao^4†^, Jianning Zhang^4†^, Siyu Lu^1,3^, Shuai Li^1,3^, Lufeng Zhao^1,3^, Ming Gao^1,3^, Hao Chen^5,6^, Hui Han^1,3*^, Xiaofang Huang^1,3*^

^1^Department of Critical Care Medicine, Qilu Hospital of Shandong University, Jinan, Shandong, China;

^2^School of Nursing and Rehabilitation, Shandong University, Jinan, Shandong, China.

^3^Innovation Research Center for Sepsis and Multiple Organ Injury, Shandong University, Jinan, Shandong, China;

^4^Department of Respiratory and Intensive Care Unit, The First Affiliated Hospital of Shandong First Medical University and Shandong Provincial Qianfoshan Hospital, Shandong Institute of Respiratory Diseases, Jinan, Shandong, China;

^5^Clinical Research Center of Shandong University, Clinical Epidemiology Unit, Qilu Hospital of Shandong University, Jinan, Shandong, China;

^6^Department of Epidemiology and Health Statistics, School of Public Health, Cheeloo College of Medicine, Shandong University, Jinan, Shandong, China.

^†^These authors contributed equally to the work.

^*^Corresponding author: Xiaofang Huang, [hxf0925@email.sdu.edu.cn;](mailto:ruif.ang@163.com;) Hui Han, [hxf0923@126.com](mailto:hxf0923@126.com;), Qilu Hospital of Shandong University, No. 107, Wen Hua Xi Road, Jinan, 250012, Shandong, China.

**Table S1. Diagnostics of stabilized inverse probability of censoring weights and artificial censoring by strategy arm.**

| **Domain** | **Metric** | **LE90** | **GT90** |
| --- | --- | --- | --- |
| Clones and censoring | Total clones, n | 218 | 218 |
|  | Used clones  (not artificially censored and with finite weights), n | 141 | 76 |
|  | Artificially censored within adherence window, % | 34.9% | 64.7% |
| Weight truncation  (stabilized weights) | Truncation thresholds (1st / 99th percentile) | 0.711, 2.042 | 0.711, 2.042 |
|  | Truncated below / above / total, % | 2.1% / 0.7% / 2.8% | 0.0% / 2.6% / 2.6% |
| Weight distribution  (raw, before truncation) | mean, median | 0.998, 0.931 | 0.999, 0.944 |
|  | 99th percentile / maximum | 1.716, 2.287 | 2.181, 2.419 |
| Effective sample size | ESS (raw / truncated) | 132.5 / 133.0 | 70.4 / 71.3 |
|  | ESS retention rate, % | 94.0% | 92.6% |
| Censoring-model behavior | Person-interval rows, n | 3906 | 2763 |
|  | Interval censoring-event rate | 1.9% | 5.1% |
|  | Probability clipping (denominator / numerator), % | 94.4%, 94.4% | 55.2%, 51.8% |

Footnote：

a. Artificial censoring was applied to enforce adherence to the prespecified treatment strategies (LE90 vs GT90) during the first 90 minutes after T_0_.

b. Stabilized IPCW was estimated using a pooled logistic model with 5-minute intervals up to 90 minutes; predicted probabilities were clipped to ε and 1−ε to avoid numerical instability.

c. Weights were truncated at the 1st/99th percentiles. ESS indicates effective sample size after truncation.

d. In the LE90 arm, a higher proportion of clipped probabilities is expected when interval-level censoring events are rare; consistent with this, stabilized weights remained close to 1 and ESS retention was high, supporting acceptable numerical stability.

e. These diagnostics assess the numerical behavior and stability of the IPCW procedure but cannot establish that all informative censoring or model misspecification has been eliminated.

f “Observed LE90 initiation group” denotes patients whose CMS initiation occurred within 90 minutes after T0. “Used clones” denotes clones that were not artificially censored and had finite IPCW estimates; this number may therefore differ from the observed initiation-group count.

Abbreviations: CMS, colistimethate sodium; ESS, effective sample size; GT90, initiation of nebulized CMS >90 min after T0; IPCW, inverse probability of censoring weighting; LE90, initiation of nebulized CMS ≤90 min after T0; T0, start of bronchodilator nebulization.

**Table S2.** **Culture surveillance intensity and follow-up sampling characteristics within 14 days (LE90 *vs.* GT90)**

| **Item** | **LE90 (n=166)** | **GT90 (n=76)** | **SMD** |
| --- | --- | --- | --- |
| Respiratory culture frequency (0–14 d) |  |  |  |
| Total respiratory cultures within 14 d, median (IQR) | 5 (4–5) | 5 (5–5) |  |
| Only 1 respiratory culture within 14 d, n (%) | 2 (1.4) | 0 (0.0) | 0.168 |
| ≥1 follow-up respiratory culture after T_0_, n (%) | 142 (100.0) | 76 (100.0) | 0.000 |
| Timing of first follow-up culture after T_0_ |  |  | 0.207 |
| ≤6 h, n (%) | 68 (47.9) | 40 (52.6) |  |
| 6–24 h, n (%) | 71 (50.0) | 36 (47.4) |  |
| 24–48 h, n (%) | 3 (2.1) | 0 (0.0) |  |
| >48 h, n (%) | 0 (0.0) | 0 (0.0) |  |
| No follow-up within 14 d, n (%) | 0 (0.0) | 0 (0.0) |  |
| First follow-up specimen type |  |  | 0.193 |
| BALF, n (%) | 56 (39.4) | 23 (30.3) |  |
| Non-BALF, n (%) | 86 (60.6) | 53 (69.7) |  |
| Bronchoscopy frequency (0–14 d) |  |  | 0.106 |
| 0, n (%) | 13 (9.2) | 5 (6.6) |  |
| 1, n (%) | 31 (21.8) | 16 (21.1) |  |
| ≥2, n (%) | 98 (69.0) | 55 (72.4) |  |
| Missing, n (%) | 0 (0.0) | 0 (0.0) |  |
| Sensitivity subset |  |  |  |
| ≥2 follow-up cultures within 7 d after T0, n (%) | 140 (98.6) | 76 (100.0) | 0.168 |

Footnotes:

a. Respiratory culture surveillance was summarized within 14 days after T0 (or until discharge/death, whichever occurred first).

b. “First follow-up culture” refers to the first respiratory culture obtained after T0 (baseline culture at/before T0 was not counted as follow-up). Patients without any follow-up respiratory culture within 14 days were categorized as “No follow-up within 14 d.”

c. Specimen type refers to the first follow-up respiratory specimen.

d. SMD is reported to quantify between-group imbalance; values <0.10 generally indicate good balance.

e. Overall, culture surveillance intensity was comparable between groups, reducing concerns that differential sampling alone explains the observed difference in microbiological clearance.

Abbreviations: BALF, bronchoalveolar lavage fluid; CMS, colistimethate sodium; GT90, initiation of nebulized CMS >90 min after T0; IPCW, inverse probability of censoring weighting; IQR, interquartile range; LE90, initiation of nebulized CMS ≤90 min after T0; SMD, standardized mean difference; T0, start of bronchodilator nebulization.

**Table S3. Sensitivity analysis using a 120-minute adherence window (LE120 *vs.* GT120) for per-protocol emulated strategies of nebulized CMS initiation after bronchodilator nebulization**

| **Outcome (0-14d)^1^** | **LE120** | **GT120** | **Effect (LE120-GT120)^2^** | ***P* value** |
| --- | --- | --- | --- | --- |
| Cumulative incidence of microbiological clearance by day 14, % (95% CI)  (death treated as a competing event) | 69.0 (61.0, 76.6) | 53.7 (40.7, 65.6) | 15.5 (0.7, 30.6) | 0.046 |
| Restricted mean time alive and  not microbiologically cleared, days (95%CI) | 7.16 (6.42, 7.95) | 9.98 (9.14, 10.89) | -2.84 (-4.01, -1.64) | <0.001 |

Footnotes

a. Sensitivity analyses were conducted by varying the adherence window defining “early” initiation as 120 minutes after T_0_, while keeping outcome definition and follow-up identical to the primary analysis (14-day microbiological clearance with death as a competing event).

b. IPCW was estimated using a pooled logistic model with 5-minute intervals up to the corresponding adherence window; probabilities were clipped at ε, and weights were truncated at the 1st/99th percentiles (primary IPCW specification).

c. An additional IPCW specification using alternative ε and alternative truncation yielded similar conclusions (Table S4).

Abbreviations: CI, confidence interval; CIF, cumulative incidence function; CMS, colistimethate sodium; GT120, initiation of nebulized CMS >120 min after T_0_; IPCW, inverse probability of censoring weighting; LE120, initiation of nebulized CMS ≤120 min after T_0_; T_0_, start of bronchodilator nebulization.

**Table S4.** Sensitivity analysis for the 90-minute adherence window (LE90 *vs.* GT90) using an alternative IPCW specification.

| **Outcome (0-14d)^2^** | **LE90** | **GT90** | **Effect (LE90-GT90)^3^** | ***P* value** |
| --- | --- | --- | --- | --- |
| CIF of microbiological clearance at day 14 (death as competing event), % (95%CI) | 71.5 (63.7, 78.3) | 52.6 (41.5, 64.0) | 18.6 (5.8, 32.8) | 0.004 |
| Restricted mean time alive and  not microbiologically cleared, days (95%CI) | 6.90 (6.21, 7.64) | 10.01(9.29, 10.87) | -3.20 (-4.27, -2.13) | <0.001 |

Footnotes:

a. This table reports results under an alternative IPCW specification to assess robustness to modeling/truncation choices.

b. Compared with the primary IPCW specification, the alternative approach used probability clipping at ε^alt^ and weight truncation at the 2.5th/97.5th percentiles; all other analysis components were unchanged (clone–censor–weight framework, 14-day competing-risk outcome, Aalen–Johansen estimation, and restricted mean time).

Abbreviations: CI, confidence interval; CIF, cumulative incidence function; CMS, colistimethate sodium; GT90, initiation of nebulized CMS >90 min after T_0_; IPCW, inverse probability of censoring weighting; LE90, initiation of nebulized CMS ≤90 min after T_0_; T_0_, start of bronchodilator nebulization.

**Table S5.** Exploratory post-clearance landmark analysis for subsequent 28-day mortality at 7 days after microbiological clearance.

| **Variable** | **Still in ICU at day 7 after clearance** | **Out of ICU by day 7 after clearance** | **Effect estimate** |
| --- | --- | --- | --- |
| Patients, n | 47 | 84 | Adjusted HR 3.35 (95% CI 1.37–8.18)；  *P* = 0.008 |
| Subsequent 28-day deaths, n (%) | 15 (31.9%) | 9 (10.7%) |  |

Footnotes: This exploratory landmark analysis was restricted to patients who achieved microbiological clearance and survived to 7 days after clearance. ICU status at the landmark was categorized as still in the ICU or out of the ICU by day 7 after clearance. Subsequent 28-day mortality was analyzed from the landmark time point using a multivariable Cox proportional hazards model adjusted for age, sex, APACHE II score, SOFA score, vasopressor use, mechanical ventilation, lactate, COPD, center, and time from T0 to microbiological clearance. Hazard ratios compare patients still in the ICU at the landmark with those already out of the ICU.

Abbreviations: CI, confidence interval; COPD, chronic obstructive pulmonary disease; HR, hazard ratio; ICU, intensive care unit; SOFA, Sequential Organ Failure Assessment. T0, start of bronchodilator nebulization.

**Table S6.** Exploratory post-clearance ICU status at 7 days after microbiological clearance according to LE90 versus GT90.

| **Outcome** | **GT90** | **LE90** | **Effect estimate** |
| --- | --- | --- | --- |
| Patients who achieved microbiological clearance, n | 41 | 106 | — |
| Still in ICU at day 7 after clearance, n (%) | 12 (29.3%) | 41 (38.7%) | Crude RD 9.4% (95% CI, -7.3% to 26.1%);  Fisher’s exact *P* = 0.341 |
| Out of ICU by day 7 after clearance, n (%) | 29 (70.7%) | 65 (61.3%) | — |

Footnotes: This exploratory analysis was restricted to patients who achieved microbiological clearance. ICU status at day 7 after clearance was categorized as still in the ICU or out of the ICU. Crude comparisons were based on observed proportions.

Abbreviations: CI, confidence interval; ICU, intensive care unit; RD, risk difference.

**Table S7.** Exploratory analysis of superinfection by day 28 according to ICU status at 7 days after microbiological clearance.

| **Variable** | **Out of ICU by day 7 after clearance** | **Still in ICU at day 7 after clearance** | **Effect estimate** |
| --- | --- | --- | --- |
| Patients, n | 84 | 47 | — |
| Superinfection by day 28, n (%) | 15 (17.9%) | 20 (42.6%) | Crude RD 24.7% (95% CI, 8.4% to 41.0%);  Fisher’s exact *P* = 0.004 |
| Multivariable logistic regression | Reference | Adjusted OR 5.51 (95% CI, 2.01–15.16); *P* < 0.001 | — |

Footnotes: This exploratory analysis was restricted to patients who achieved microbiological clearance and survived to 7 days after clearance. ICU status at day 7 after clearance was categorized as still in the ICU or out of the ICU. Because exact timestamps for superinfection were unavailable, superinfection by day 28 was analyzed as a binary outcome rather than as a formal landmark time-to-event endpoint. Adjusted odds ratios were estimated using multivariable logistic regression adjusting for age, SOFA score, vasopressor use, mechanical ventilation, COPD, center, and time from T0 to microbiological clearance.

Abbreviations: CI, confidence interval; COPD, chronic obstructive pulmonary disease; ICU, intensive care unit; OR, odds ratio; RD, risk difference; SOFA, Sequential Organ Failure Assessment; T0, start of bronchodilator nebulization.

**Figure S1.** **Covariate balance before and after IPCW in the per-protocol emulation.** Absolute standardized mean differences (|SMD|) for prespecified baseline covariates are shown for the unweighted sample (triangles) and after applying stabilized inverse probability of censoring weights (IPCW; circles) used in the clone–censor–weighting analysis. Smaller |SMD| values indicate better balance. The dashed vertical line marks |SMD| = 0.10 (commonly used threshold for acceptable balance). Covariates include age, sex, BMI, APACHE II, SOFA, center, PaO_2_/FiO_2_, lactate, HAP/VAP status, nebulizer device type, vasopressor use, mechanical ventilation (MV), COPD, and concomitant intravenous colistin.

Abbreviations: APACHE II, Acute Physiology and Chronic Health Evaluation II; BMI, body mass index; COPD, chronic obstructive pulmonary disease; HAP, hospital-acquired pneumonia; IPCW, inverse probability of censoring weighting; MV, mechanical ventilation; PaO_2_/FiO_2_, ratio of arterial oxygen partial pressure to fraction of inspired oxygen; SMD, standardized mean difference; SOFA, Sequential Organ Failure Assessment; VAP, ventilator-associated pneumonia.

**Figure S2.** **Diagnostics of IPCW by strategy arm.** (A) Boxplots of stabilized IPCW before (raw) and after truncation, shown on the log10 scale. (B) Kernel density of stabilized IPCW before and after truncation, with dashed lines indicating truncation thresholds. Predicted interval-level censoring probabilities were bounded to (0.05, 0.95) (ε=0.05), and stabilized weights were truncated at the 1st and 99th percentiles. ESS indicates effective sample size under weighting.

Abbreviations: ESS, effective sample size; CMS, colistimethate sodium; GT90, initiation of nebulized CMS >90 min after T0; IPCW, inverse probability of censoring weighting; LE90, initiation of nebulized CMS ≤90 min after T_0_; T_0_, start of bronchodilator nebulization.
